# Estimation of kidney function in patients with primary neuromuscular diseases: is serum cystatin C a better marker of kidney function than creatinine?

**DOI:** 10.1007/s40620-021-01122-x

**Published:** 2021-08-05

**Authors:** Annika Aldenbratt, Christopher Lindberg, Elias Johannesson, Ola Hammarsten, Maria K. Svensson

**Affiliations:** 1 Department of Medical Sciences, Uppsala University, Uppsala, Sweden; 2 Neuromuscular Center/Department of Neurology, Sahlgrenska University Hospital, Gothenburg, Sweden; 3 Department of Psychology, Pedagogy and Sociology, University West, Trollhättan, Sweden; 4 Department of Clinical Chemistry, Sahlgrenska Academy, Gothenburg, Sweden

**Keywords:** Cystatin C, Creatinine, Estimated GFR, Iohexol clearance, Muscle mass, Neuromuscular disease

## Abstract

**Background:**

Using serum creatinine leads to an overestimation of kidney function in patients with primary neuromuscular disorders, and reduced kidney function may remain undetected. Cystatin C (CysC) could provide a better estimation.

**Aim:**

To evaluate the precision, accuracy, and bias of two creatinine-, one cystatin C-based and one combined equation to estimate glomerular filtration rate (eGFR) in patients with primary neuromuscular disease.

**Patients and methods:**

Of the 418 patients initially identified at the out-patient clinic, data on kidney function was obtained for 145 adult patients (age 46 ± 14 years, BMI 26 ± 6 kg/m^2^) with primary neuromuscular disease. Kidney function was measured by iohexol clearance, and blood samples for serum creatinine and CysC were drawn simultaneously. Bias was defined as the mean difference between eGFR and measured iohexol clearance, and accuracy as the proportion of eGFRs within ± 10% (P10) of measured clearance.

**Results:**

Kidney function (iohexol clearance) was 81 ± 19 (38–134) ml/min/1.73m^2^. All equations overestimated kidney function by 22–60 ml/min/1.73m^2^. eGFR CysC had the lowest bias overall 22 (95% CI 20–26) ml/min/1.73m^2^ also at all levels of kidney function we evaluated (at 30–59 ml/min/1.73m^2^ bias was 27 (95% CI 21–35), at 60–89 it was 25 (95% CI 20–28) and at ≥ 90 it was 12 (95% CI 7–22)). eGFR CysC also had the best accuracy in patients with reduced kidney function (P10 was 5.9% at 30–59 ml/min/1.73m^2^).

**Conclusions:**

Cystatin C-based estimations of kidney function performed better than creatinine-based ones in patients with primary neuromuscular disease, but most importantly, all evaluated equations overestimated kidney function, especially in patients with reduced kidney function. Therefore, kidney function should be measured by gold-standard methods when precision and accuracy are needed.

**Graphic abstract:**

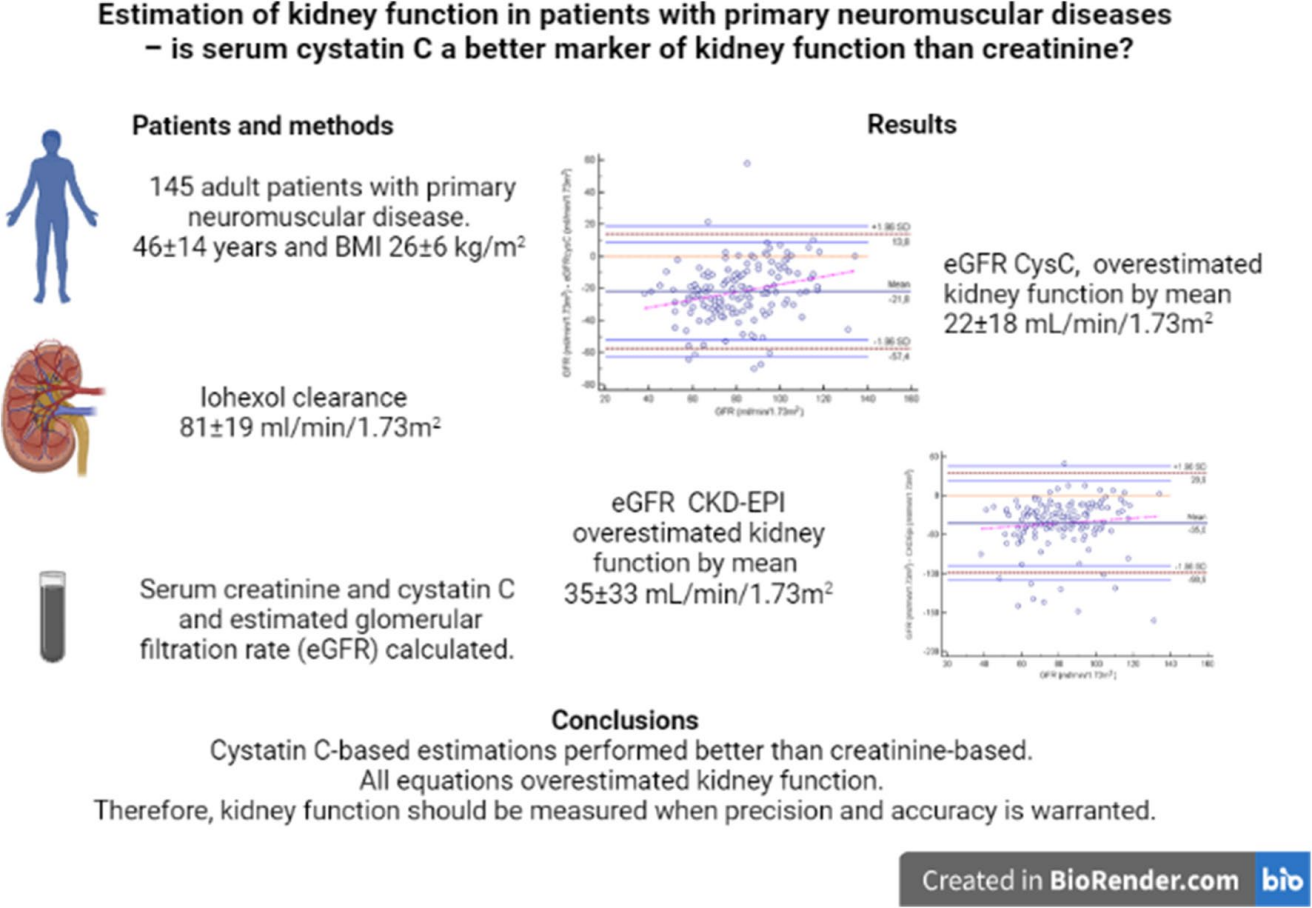

**Supplementary Information:**

The online version contains supplementary material available at 10.1007/s40620-021-01122-x.

## Introduction

Glomerular filtration rate (GFR) is a validated marker of kidney function [1, 2] recommended in treatment guidelines [3, 4]. In patients with neuromuscular diseases muscle mass is diminished and therefore creatinine-based estimations systematically overestimate kidney function and reduced kidney function may remain undetected and undiagnosed [5, 6]. When serum creatinine is combined with information on lean body mass, a slightly better estimate is achieved [7]. Cystatin C (CysC) correlates well with the clearance of inulin, ^51^Cr-EDTA and iohexol, and is not affected by muscle mass to the same extent as creatinine [8, 9] and has therefore been suggested as a better marker of kidney function [10–12]. CysC has been evaluated in different patient populations, but few studies have assessed CysC in patients with primary neuromuscular diseases including Duchenne muscular dystrophy (DMD) [13, 14], amyotrophic lateral sclerosis (ALS) [15] and myotonic dystrophy type 1 (DM1) [16]. Recent studies have shown that CysC concentrations are affected by determinants other than kidney function and muscle mass. Age, male gender, body mass index (BMI), fat mass, triglycerides, hypertension, uric acid, C-reactive protein and diabetes have all been associated with higher serum concentrations of CysC regardless of kidney function [12, 17]. This is of importance since patients with muscular dystrophy often have an absolute or relative increase in body fat [18, 19]. Treatment with high doses of glucocorticoids, as well as thyroid dysfunction and ethnicity also influence CysC levels [10–12]. A report from the Swedish Council on Health Technology Assessment highlighted that cystatin C-based equations had not been sufficiently evaluated in patients with a low BMI [20].

This study was designed to evaluate the precision, accuracy, and bias of two creatinine-, one cystatin C-based and one combined equation to estimate glomerular filtration rate (eGFR) in patients with primary neuromuscular diseases. The findings may also be valuable for subjects with low muscle mass or BMI due to other reasons [21].

## Patients and methods

### Patients

**Fig. 1:**
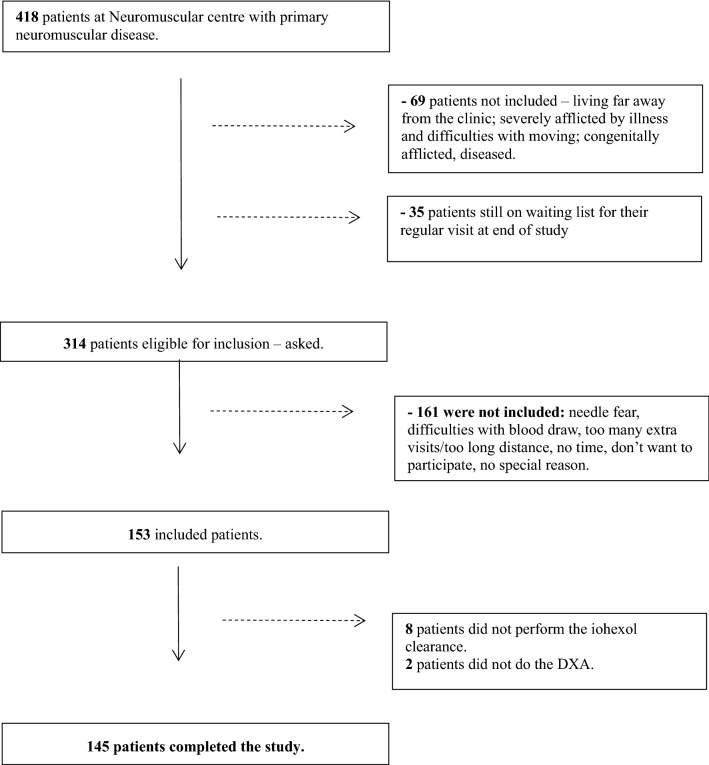
A flowchart of the recruitment of study participants

Out of 418 adult patients diagnosed with primary neuromuscular diseases at the Neuromuscular Centre at Sahlgrenska University Hospital, Gothenburg, 314 were asked to participate in the study, 153 patients accepted to participate and finally 145 patients (68 men and 77 women) were included in the study and their kidney function was evaluated. A flowchart describing the recruitment of study participants is shown in Fig. 1. The majority of patients (n = 94) had DM1, 19 had Facioscapulohumeral muscular dystrophy (FSHD), 15 had Limb-girdle muscular dystrophy (LGMD), eleven had Spinal muscular atrophy (SMA), three had DMD and three had Becker muscular dystrophy (BMD). Inclusion criteria were: age above 18 years and having a primary neuromuscular disease. The reduction in muscle function ranged from near-normal strength to severe weakness, not excluding wheelchair users and patients with need of assisted ventilation with Bilevel Positive Airway Pressure (BiPAP), however, patients with severe muscle contractures making the study examinations difficult or impossible were excluded. Exclusion criteria were: estimated survival less than 1 year, known ischemic heart disease, malignancy or an inadequately treated endocrine disease. In addition, included patients should not have a previous history of kidney disease or reduced kidney function. Two patients receiving ongoing medication with corticosteroids were not excluded. Patients were included in the study between 1st October, 2010 and 31st January, 2014. The study was approved by the ethics committee of Gothenburg, Sweden (dnr 492-10). Study procedures were performed according to the principles of the Declaration of Helsinki and written informed consent was obtained from all participants.

### Methods

Blood samples for analysis of serum creatinine and cystatin C were drawn simultaneously as iohexol plasma-clearance was performed. Measured kidney function (clearance) was determined by plasma-clearance of iohexol (Bis(2,3-dihydroxypropyl)-5-[N-(2,3-dihydroxypropyl)-acetamido]-2, 4,6-triiodo-isophthalamide) according to routine procedures. Five.0 mL of iohexol (Omnipaque® 300 mg J/mL, Nycomed) was administered intravenously. After 4 h a second blood sample was drawn from the contralateral arm for analysis of iohexol concentration. The distribution volume was estimated using a function related to body weight, and another method was used to correct for the lack of complete uniform distribution and non-immediate mixing. The analysis and calculations of iohexol clearance were performed at the Department of Clinical Chemistry at Sahlgrenska University Hospital/Mölndal. This department participates in inter-laboratory comparison schemes from Equalis and has been certified (https://www.equalis.se/en/). Iohexol concentration was determined by HPLC [22, 23] with a between-day Coefficient of variation (CV) of 2.6%. IDMS-calibrated serum creatinine and cystatin C concentrations were analysed using a Cobas system (Roche) at the Department of Clinical Chemistry at Sahlgrenska University Hospital. TheCV for the creatinine and Cystatin C methods was less than 5% within the range in this patient population.

The indexed eGFRs (ml/min/1.73 m^2^) were calculated using the following equations:

- MDRD (175 × SCr^−1,154^ + age^0.203^ + 1.212 (if patient is black) × 0.742 (if female) [24]
- CKD-EPI (141 × min (SCr/k, 1)^a^ × max (SCr/k, 1)^−1,209^ × 0.993^age^ × 1.018 (if female);
- (a = − 0.329 if female, a = − 0.411 if male)) [25]
- Cystatin C-based eGFR (eGFR CysC)130 × cystatin C^−1.069^ × age^−0.117^ − 7 [26]
- Combined mean value cystatin C- and creatinine-based eGFR (eGFR CysC + CKD-EPI)

Unindexed kidney function (clearance) and eGFRs (ml/min) were calculated using the following equation:

Absolute GFR (ml/min) = relative GFR [ml/min/body surface area (1.73 m^2^)] × body surface area (m^2^)/1.73(m^2^) and the body surface area (BSA) was calculated with the DuBois and DuBois formula; body area (m^2^) = 0.20247 × length (m) 0.725 × weight (kg) 0.425 [27].

### Statistical methods

Continuous data variables are expressed as mean (SD) if parametric and as median (interquartile range (IQR)) if non-parametric using the Shapiro-Wilks test for normality. Correlations between estimations (eGFR) and measured clearance were performed using both bivariate and multivariable Spearman's correlation analysis. ρ in text and tables is the Spearman's rank correlation coefficient. In multivariable analyses, adjusting for gender, age, smoking status and muscle mass [skeletal muscle index; (SMI)], partial Spearman's Rho method was used and ρ is the partial correlation coefficient. Bias was assessed as the mean difference (eGFR-measured clearance), with negative values indicating lower eGFR than measured kidney function (underestimation) and positive values indicating overestimation. Accuracy was defined as the proportion of eGFRs within ± 30% (P30) and ± 10% (P10) of measured clearance. The 95% confidence intervals (95% CIs) were calculated as measures of the statistical uncertainty when estimating bias and accuracy using bootstrap with 1000 replications, which gives good estimates of confidence intervals. Bias and accuracy P30 and P10 were evaluated in relation to the kidney function levels 30–59, 60–89 and ≥ 90 ml/min/1.73m^2^ (26). The significance of the differences between eGFR equations was determined with the paired sign test for the bias. McNemar's exact test for correlated proportions P30 and P10 was used for pairwise comparisons. Bland–Altman diagrams were used to depict bias and the limits of agreement [28]. In these diagrams, the reference method, e.g. measured clearance (rather than the mean of the two methods) is shown on the x-axis. This approach has been recommended when one of the two methods can be considered more accurate [29], but this has also been challenged by Stevens et al. since clearance too is measured with error. A p value of ≤ 0.05 was considered as significant. MedCalc (MedCalc Software, Broekstaat 52, 9030 Mariakerke, Belgium) and IBM SPSS Statistics 22.0 (IBM Corporation, 1 New Orchard Road, Armonk, New York 10504-1722, U.S.) software were used for the analyses.

## Results

### Study participants

Clinical and biochemical characteristics of the study participants (n = 145) are shown in Table 1. The overall kidney function (measured clearance) was 81 ± 19 (38–134) ml/min/1.73m^2^ with no difference between men and women (not shown). Only 18 patients had a measured clearance below 60 ml/min/1.73m^2^. Unindexed kidney function (measured clearance) and eGFRs are shown in Supplementary Table 1.

**Table 1:** Clinical and biochemical characteristics of study participants (n = 145)

	Different levels of measured kidney function (clearance) (ml/min/1.73 m^2^)				
	All (n = 145)	30–59 (n = 18)	60–89 (n = 79)	≥ 90 (n = 48)	p-value^a^
Age (years)	46 (14)	57.7 (10)	47.6 (13)	39.7 (13)	< 0.001
Length (cm)	171.5 (9.8)	170.2 (7.0)	171.7 (9.5)	170.6 (10.8)	0.75
Weight (kg)	76.8 (18.3)	73.2 (18.6)	79.2 (19.7)	72.8 (15.7)	0.12
BMI (kg/m^2^)	25.9 (6.2)	24.8 (5.1)	26.7 (7.7)	24.9 (5.0)	0.26
Gender					
Male *n* (%)	68 (46)	8 (12)	37 (54)	23 (34)	0.97
Female *n* (%)	77 (54)	10 (54)	42 (54)	25 (32)	0.97
SMI (kg/m^2^)	6.2 (2.1)	5.6 (2.6)	6.6 (2.4)	5.9 (3.1)	0.13
S-cystatin C (mg/L)	0.96 (0.2)	1.2 (.2)	.99 (.1)	.86 (.1)	< 0.001
S-creatinine (ųmol/L)	58.2 (23.8)	65.6 (26.9)	63.1 (21.7)	45.7 (21.6)	0.03
MDRD (ml/min/1.73m^2^)	113 (69.3)	101.5 (60.8)	100.0 (43.9)	152.5 (159.4)	< 0.001
CKD-EPI (ml/min/1.73m^2^)	111.0 (29.0)	99.5 (41.8)	106.0 (27.0)	129.0 (33.5)	< 0.001
eGFR CysC (ml/min/1.73m^2^)	104.7 (25.6)	82.3 (19.4)	98.1 (19.3)	118.3 (19.3)	< 0.001
eGFR CysC + CKD-EPI (ml/min/1.73m^2^)	111.8 (27.9)	91.1 (27.0)	103.7 (22.1)	121.2 (23.5)	< 0.001
Diagnosis (%)					
Myotonic dystrophy 1	94 (64.8)	16 (17)	64 (68)	14 (15)	
Duchenne and Becker muscular dystrophy	6 (4)	1 (17)	2 (33)	3 (50)	
Facioscapulohumeral	19 (13)	0 (0)	4 (21)	15 (79)	
Muscular dystrophy (FSHD)					
Limb-girdle muscular dystrophy	19 (13)	1 (5)	8 (42)	10 (53)	
Spinal muscular atrophy (SMA)	7 (5)	0 (0)	1 (14)	6 (86)	

Results expressed as mean ± SD. Diagnosis expressed as *n* (%)

*SMI* skeletal muscle index; *FSHD* Facioscapulohumeral muscular dystrophy; *Limb-girdle* Limb-girdle muscular dystrophy *SMA* spinal muscular atrophy, *eGFR* estimated GFR, *CKD-EPI* chronic kidney disease epidemiology collaboration; *MDRD* modification of diet in renal disease [1, 31]

### Correlations between measured clearance and estimated glomerular filtration rates (eGFRs)

The correlations between BSA-indexed measured clearance and eGFRs are shown in Table 2. All eGFRs correlated to measured clearance (ρ = 0.41–0.64) and the strongest correlation was found between the cystatin C-based eGFR (eGFR CysC) (ρ = 0.64) followed by the combined cystatin C- and creatinine–based eGFR (eGFR CysC + CKD-EPI) (ρ = 0.61) and the creatinine-based eGFR (CKD-EPI) (ρ = 0.48). The correlations for these three equations remained significant after adjusting for age, gender, smoking status and SMI (ρ = 0.52, ρ = 0.46 and ρ = 0.45). The MDRD equation displayed the largest change after adjustment and the correlation to measured clearance was markedly reduced from ρ = 0.41 to ρ = 0.11 (NS). When unindexed measured clearance and GFR estimates (ml/min) were used, correlations were similar, except for unadjusted MDRD that was markedly reduced (ρ = 0.12) and non-significant. After adjustment, MDRD improved slightly to ρ = 0.12 and became significant (see Supplementary Table 2).

**Table 2:** Correlations between estimated (eGFR) and measured kidney function (clearance) (n = 145)

	Unadjusted clearance		Adjusted clearance^a^	
MDRD (ml/min/1.73m^2^)	0.41	(0.25, 0.55)	0.11	(− 0.07, 0.32)
CKD-EPI (ml/min/1.73m^2^)	0.48	(0.33, 0.62)	0.45	(0.10, 0.45)
eGFR CysC (ml/min/1.73m^2^)	0.64	(0.51, 0.73)	0.52	(0.38, 0.64)
eGFRCys C + CKD-EPI (ml/min/1.73m^2^) 0.61	(0.47, 0.71)	0.46	(0.28, 0.62)	

95% confidence intervals (95% CI) based on bootstrap in parentheses. All correlations except adjusted MDRD were significant, p < 0.05

*MDRD* modification of diet in renal disease study group, *CKD-EPI* chronic kidney disease epidemiology collaboration, *eGFR CysC* Cystatin C-based estimated GFR, *eGFR* CysC + CKD-EPI; combined Cystatin C-based and CKD-EPI (mean values), see methods section

^a^Adjusted for age, gender, smoking status and muscle mass (SMI, skeletal muscle index)

### Bias for estimated glomerular filtration rates (eGFRs)

Table 3 compares bias and accuracy of BSA-indexed eGFRs according to level of kidney function (clearance). With regard to overall bias, all equations overestimate kidney function, from 22 to 60 ml/min/1.73m^2^, with eGFR CysC having the smallest bias (22), followed by CKD-EPI (27), MDRD (32) and eGFR CysC + CKD-EPI (26) (all p < 0.05). When comparing bias at different kidney function levels different patterns were found for the equations. eGFR CysC had the lowest bias at all levels of kidney function (clearance) and overall eGFR CysC had a significantly lower bias than MDRD and eGFR CysC + CKD-EPI. All equations had a larger bias in patients with reduced kidney function, i.e. measured clearance below 60 ml/min/1.73m^2^.

**Table 3:** Comparison of the performance (bias and accuracy) of estimated GFR equations (eGFR) by kidney function (clearance), overall and at different levels of kidney function (n = 145)

	Different levels of kidney function (clearance) (ml/min/1.73m^2^)			
	Overall (n = 145)	30–59 (n = 18)	60–89 (n = 79)	≥ 90 (n = 48)
Bias^a^ (ml/min/1.73 m^2^)				
MDRD	31.7 (22.9, 39.2)*	45.5 (15.5, 65.0)*	28.0 (18.0, 35.6)	48.1 (25.1 82.5)*
CKD-EPI	27.0 (24.0, 35.0)	45.0 (20.0, 57.0)*	30.0 (24.0, 36.0)*	23.5 (15.0, 39.0)*
eGFR CysC	22.2 (19.1, 25.2)	27.2 (20.7, 35.1)	25.2 (19.8, 28.4)	11.9 (7.4, 21.8)
eGFR CysC + CKD-EPI	26.1 (23.6, 29.1)*	34.3 (25.4, 49.1)*	27.2 (24.1, 30.2)*	19.2 (16.5, 25.0)*
Accuracy (P10)^b^ (%)				
MDRD	15.9 (10.3, 22.1)	5.6 (0.0, 18.8)	19.0 (10.9, 27.5)	14.6 (5.1, 25.6)*
CKD-EPI	11.0 (6.2, 16.6)*	0.0 (0.0, 0.0)	7.6 (2.6, 14.3)	20.8 (9.5, 32.7)*
eGFR CysC	20.8 (13.9, 27.8)	5.9 (0.0, 20.0)	7.6 (2.4, 13.8)	47.9 (34.0, 61.7)
eGFR CysC + CKD-EPI	11.8 (6.9, 18.1)*	0.0 (0.0, 0.0)	5.1 (1.2, 10.4)	27.1 (15.1, 40.0)*
Accuracy (P30) ^b^ (%)				
MDRD	38.6 (31.1, 46.9)	22.2 (4.5, 43.8)	41.8 (30.1, 52.3)	39.6 (25.6, 52.9)*
CKD-EPI	37.2 (29.7, 45.5)*	11.1 (0.0, 27.8)	31.6 (21.1, 42.2)	56.3 (40.5, 69.4)*
eGFR CysC	49.3 (41.0, 56.9)	11.8 (0.0, 28.6)	38.0 (27.2, 48.7)	81.3 (68.9, 91.1)
eGFR CysC + CKD-EPI	43.8 (35.4, 51.4)	5.9 (0.0, 19.0)	34.2 (23.4 45.0)	72.9 (58.8, 84.3)

*CKD-EPI* chronic kidney disease epidemiology collaboration; *MDRD* modification of diet in renal disease

*p ≤ 0.05 vs eGFR CysC

^a^Bias was assessed the mean difference (eGFR-measured clearance) with negative values indicating lower eGFR than measured clearance (underestimation of kidney function) and positive values indicating overestimation

^b^Accuracy is defined as the proportion of eGFRs within ±  > 30% (P30) and within ±  > 10% (P10) of measured clearance (95% CI)

Accuracy was evaluated as measured clearance ± 10% (P10) and ± 30% (P30) and the overall accuracy for the different equations varied, P10 from 5.6 to 21% and P30 from 20 to 49%. Overall eGFR CysC had a significantly higher overall P10 than MDRD and eGFR CysC+CKD-EPI*.* When comparing the accuracy at different levels of kidney function MDRD and eGFR CysC had a low P10 in patients with reduced kidney function (5.6 and 5.9%, respectively). At P30 all creatinine-based equations improved their performance overall but the 95% CIs were wide. Among the creatinine-based equations, MDRD had the best performance in patients with reduced kidney function (22%) and CKD-EPI in patients with normal kidney function (56%). eGFR CysC had the best accuracy in patients with normal kidney function, significantly higher than both MDRD and eGFR CKD-EPI but the P30 in patients with reduced kidney function was only 12%. Unindexed kidney function (measured clearance) and eGFRs are shown in Supplementary Table 3 and commented on below.

### Bland–Altman diagrams for estimated glomerular filtration rates (eGFRs)

**Fig. 2:**
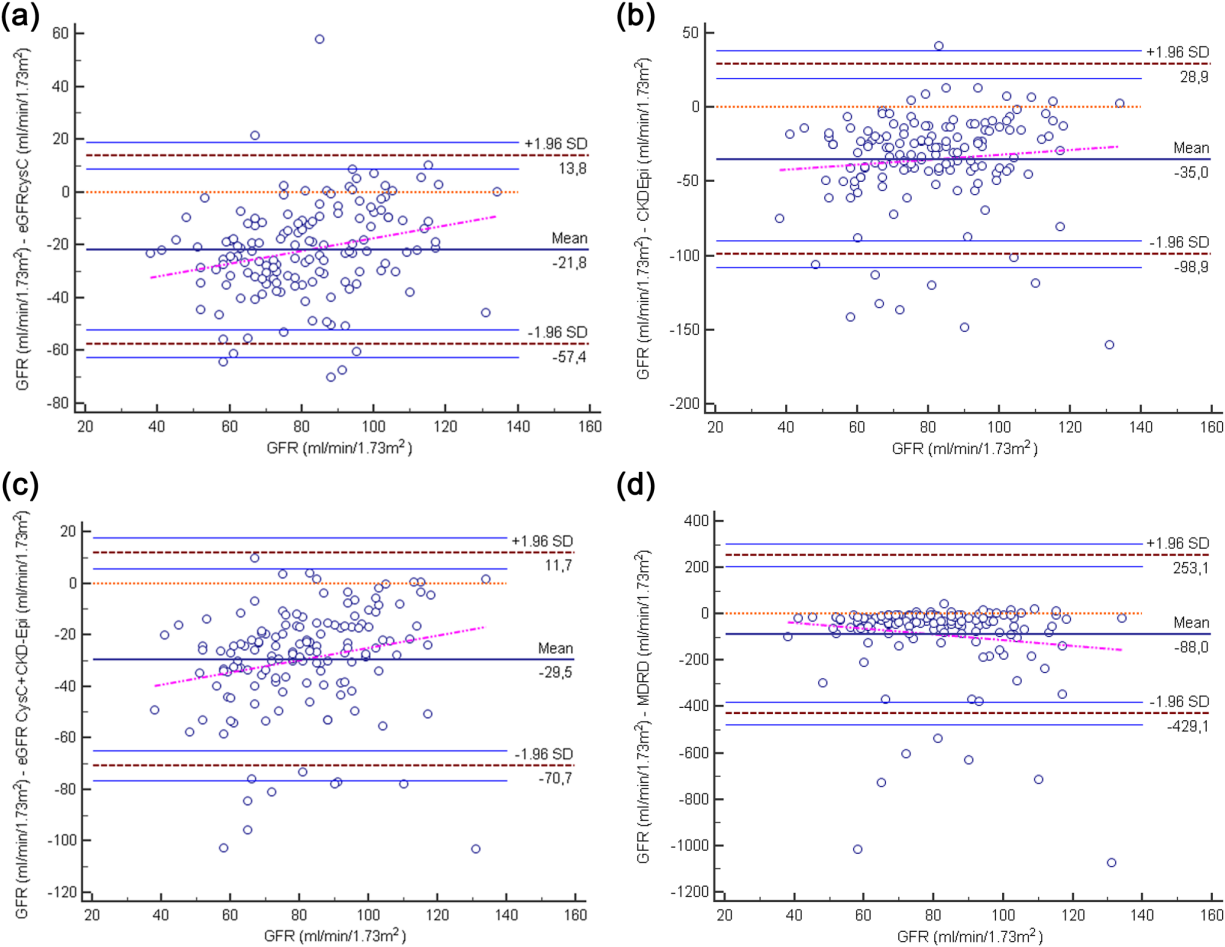
**a** Bland–Altman-plots of the differences between estimated GFR (eGFR Cys C) and measured iohexol clearance. Kidney function (measured clearance) levels as indicated; 38–59, 60–89 and ≥ 90 ml/min/1.73m^2^. **b** Bland–Altman-plots of the differences between estimated GFR (eGFR CKD-EPI) and measured clearance. Kidney function (measured clearance) levels as indicated; 38–59, 60–89 and ≥ 90 ml/min/1.73m^2^. **c** Bland–Altman-plots of the differences between (eGFR CysC + CKD-EPI) and measured iohexol clearance. Kidney function (measured clearance) levels as indicated; 38–59, 60–89 and ≥ 90 ml/min/1.73m^2^. **d** Bland–Altman-plots of differences between (eGFR MDRD) and measured iohexol clearance. Kidney function (measured clearance) levels as indicated; 38–59, 60–89 and ≥ 90 ml/min/1.73m^2^.*GFR* measured iohexol clearance

The bias for different equations are also illustrated by Bland–Altman diagrams displayed in Fig. 2a–d. eGFR CysC, CKD-EPI, eGFR CysC + CKD-EPI and MDRD overestimated kidney function; GFR (measured clearance) by mean 22 ± 18, 35 ± 33, 30 ± 21 and 88 ± 174 ml/min/1.73m^2^, respectively. Thus, MDRD exhibited the largest bias. The largest overestimations using eGFRs were found in patients with reduced kidney function. Figures using unindexed kidney function (measured clearance) and eGFRs are found in Supplementary Fig. 2a–d.

### Summary of comparisons between BSA-indexed and unindexed values evaluating bias and accuracy

Overall, only minor changes were observed when unindexed measured clearances, i.e. absolute values (ml/min) were used instead of BSA-indexed and eGFRs, i.e. relative values (ml/min/1.73m^2^). Important to note is that the use of unindexed measured clearances and eGFRs also resulted in a change in number of subjects in the different kidney function-level categories, in 30–59 ml/min 18 to 16, in 60–89 ml/min 79–61 and in > 90 ml/min 48–67. When using unindexed measured clearance and eGFRs the overall difference in bias between eGFR CysC and eGFR CKD-EPI and the differences in accuracy between P10 eGFR CysC and eGFR CysC + CKD-EPI, and P30 eGFRCys and eGFR CysC + CKD-EPI, respectively, became significant. Figures and tables using unindexed measured clearances and eGFRs are found in the Supplementary section.

## Discussion

In this study we show that s using a cystatin C-based equation to estimate kidney function in patients with primary neuromuscular disease results in better precision, accuracy and lower bias compared to creatinine-based estimations, but most importantly, we show that all creatinine- and cystatin C-based equations evaluated in this study overestimate kidney function in this patient population and especially in patients with reduced kidney function. In addition, and in contrast to other patient populations, a combined equation based on the mean values of creatinine and cystatin C is not more precise and accurate than a cystatin C-based estimation [30].

Previous studies have demonstrated the potential usefulness of cystatin C as a marker of kidney function in patients with Duchenne muscular dystrophy [13, 14], amyotrophic lateral sclerosis [15] and myotonic dystrophy 1 [16], but in a study in children and adolescents with spinal dysraphism the authors concluded that slightly to moderately reduced kidney function could still remain undiagnosed [31]. Interestingly, in a recent study in community-dwelling older adults with sarcopenia and chronic kidney disease eGFR creatinine was not significantly correlated to muscle volume and strength, on the contrary, eGFR cystatin C was positively correlated to these parameters [32]. The findings in our study support guidelines recommending cystatin C-based estimation of kidney function when creatinine-based equations could be inaccurate. However, a weakness with the current guidelines is that they do not specify in which populations this should be done [3, 4]. Even though a cystatin C-based equation may provide better accuracy, in our study it varies within a wide range especially in patients with reduced kidney function (35–67%). In addition, a bias of 22 and a low P30 of 49% could probably not be considered “good enough” for the use of these estimates in clinical practise, especially in patients where a reduction in kidney function is suspected. We therefore argue that kidney function should be measured and not just estimated in patients in whom better accuracy is warranted, i.e. when drug dosing is important and especially when renally excreted and potentially toxic drugs and contrast media are administered.

A clinically important finding in this study is that both creatinine- and cystatin C-based equations systematically overestimate kidney function in patients with primary neuromuscular diseases, especially in patients with reduced kidney function. The reason for this overestimation when using cystatin C-based estimates is not entirely clear since previous studies have shown that cystatin C is less correlated to muscle mass and diet than creatinine [33, 34]. Previously identified non-GFR-related factors such as inflammation, obesity [17], insulin resistance [35, 36], oxidative damage [37], growth hormone [38], thyroid hormone [39] and glucocorticoids [10] have all instead been linked to increased cystatin C production and thus an underestimation of GFR. A potential explanation for the overestimation of kidney function when using cystatin-C based equations could be that body fat is a determinant of cystatin C [12] and that patients with muscular dystrophy would have not only a reduction in muscle mass but also in fat mass. This could be true but might not be the entire explanation since many patients with muscular dystrophy actually have either an absolute or a relative increase in body fat [18, 19]. To our knowledge no factor resulting in a true decreased production of cystatin C, and hence in an overestimation of kidney function, has been reported.

The convention of indexing glomerular filtration rate to BSA attempts to normalize kidney function across populations of differing body size but may be inappropriate when a more precise estimation of kidney function is needed or in patients with extreme body sizes. Adding to the complexity, there is a disproportionate relation between extracellular volume (ESV) and BSA [40], and a higher ECV in women may be concealed by scaling to BSA [41]. The use of absolute values has been shown to improve the performance of estimation of individual kidney function [42]. We therefore explored the use of both indexed and unindexed measures and estimates of kidney function in these subjects with muscle wasting diseases. In our study only minor changes were observed when unindexed values were used instead of BSA-indexed ones. One potential reason is that the patient population in this study, despite having muscle wasting conditions, did not display extreme body size. They also had a relatively normal BSA (1.88 m^2^) close to the BSA used for normalization (1.73 m^2^) of kidney function.

A weakness when using cystatin C-based estimations of kidney function has previously been the lack of an international calibrator resulting in analytical bias and inability to compare cystatin C analysis carried out using different assays. Recently, an international cystatin C calibrator was used to develop an assay-independent cystatin C-based equation for estimation of GFR (CAPA) [26]. It is however worth pointing out that CAPA does not have a sex coefficient and a potential impact of sex has not been evaluated in this study. In this study cystatin C was calibrated and the matching CAPA equation was used. This has not been done previously in studies with patients with low muscle mass. Interestingly, in this population, a combined equation based on the mean value of creatinine- and cystatin C-based estimates was not more precise and accurate than a cystatin C-based estimation; something that has been shown in other populations [30]. A plausible explanation for this finding is the overestimation of kidney function for both creatinine- and cystatin C-based estimations. Among the creatinine-based equations evaluated, MDRD had the best accuracy in patients with reduced kidney function, both for P30 and to a lesser extent also P10. MDRD is currently the most commonly used creatinine-based equation in subjects with reduced kidney function.

The findings of our study should however be interpreted in light of its strengths and limitations. The strengths include that the study population is larger than in previous corresponding studies, the measurements of serum creatinine and cystatin C were done using standardized and calibrated assays, and the analysed blood samples were drawn simultaneously with the measurements of iohexol clearance. However, there are also some limitations. One is that we pooled patients with different primary neuromuscular diseases who may have had different disease characteristics, and that from the initial 418 patients identified at the outpatient clinic data on renal function was obtained only in 145 cases in CKD stage I–IIIa, thus few participants had severe kidney failure or an advanced neuromuscular disease with very low muscle mass. The generalizability to all patients with reduced muscle mass should thus be further evaluated. The calibration of measured and estimated kidney function by BSA has been questioned in patients with altered body composition, i.e. both low and high body mass index, however, this concern is not specific for this study [40, 43] but nonetheless, we have tried to address this issue by calculating both indexed and unindexed measures and estimates.

Another limitation and potential concern is that plasma clearance of iohexol used to measure kidney function in this study has not been validated specifically in patients with primary neuromuscular disease or in other populations with low body mass index and reduced muscle mass, i.e. sarcopenia, but there are studies ongoing to assess this matter (personal communication).

Another potential explanation for part of the results in this study may thus be that both the MDRD and the CKD-EPI equation were developed using urinary clearance of iothalamate as a measurement of kidney function; GFR. This is in contrast with our study but also in the development of the cystatin C -based estimation using CAPA where plasma clearance of iohexol was used. A recent paper has shown that there may be an approx. 15% difference between urinary clearances for the two tracers [44]. Since plasma clearance is thought to be greater than urinary clearance, plasma clearance for iohexol may be 5–10% lower than urinary clearance for iothalamate, therefore, this must be taken into account.

It should also be highlighted that Pearson's correlation coefficient does not take into account any differences in values between compared groups and thus the correlation can be quite good even when eGFR values differ from measured clearance values by twofold or more. To explain the difference between agreement and correlation it is therefore important to depict bias and the limits of agreement using Bland–Altman diagrams.

In conclusion, our findings indicate that a cystatin C-based estimation of kidney function may be more accurate in patients with primary neuromuscular disease and low muscle mass. However, even though it provides better accuracy, it varies within a wide range and should thus not be considered good enough for use in patients in whom better accuracy is warranted, for example when contrast media and potentially toxic drugs are administered.

Another important finding is that both creatinine- and cystatin c-based equations systematically overestimated kidney function, especially in patients with reduced kidney function. This is clinically relevant, since detecting and diagnosing patients with reduced kidney function is extremely important as they are at risk of developing secondary metabolic  complications and/or end-stage kidney disease, and the reduction of kidney function may affect drug dosing. Further studies are necessary both to validate currently used techniques for measuring kidney function in patients with low body mass index and reduced muscle mass, as well as to evaluate diagnostic strategies for estimating kidney function in patients with reduced muscle mass and altered body composition.

## Supplementary Information

Below is the link to the electronic supplementary material.

Supplementary file1 (DOCX 204 KB)

## Abbreviations

eGFR: Estimated glomerular filtration rate
CKD-EPI: Chronic kidney disease epidemiology collaboration
MDRD: Modification of diet in renal disease study group
IDMS: Isotope dilution mass spectrometry
BMI: Body mass index
