# Supplementary material for: Estimation of kidney function in patients with primary neuromuscular diseases: is serum cystatin C a better marker of kidney function than creatinine?
Source: J Nephrol. 2021 Aug 5;35(2):493–503. doi: 10.1007/s40620-021-01122-x (PMC8926948; doi:10.1007/s40620-021-01122-x)
Supplement: Supplementary file 1 — Supplementary file1 (DOCX 204 KB) [file 40620_2021_1122_MOESM1_ESM.docx]

**Supplementary Table 1.**

Clinical and biochemical characteristics of study participants (n=145).

**Different levels of kidney function (clearance) (mL/min)**

**All (n=145) 30-59 (n=16) 60-89 (n=61) ≥90 (n=67) p-value^a^**

Age (years) 46 (13.6) 55.0 (11.9) 49.1 (14.0) 41.9 (12.3) <0.001

Length (cm) 171.1 (9.6) 165.0 (6.2)* 170.6 (9.4) 173.3 (9.9)* <0.001

Weight (kg) 76.3 (18.5) 60.1 (12.7)* 74.7 (16.5)* 82.4 (18.2)* <0.001

BMI (kg/m^2^) 25.9 (5.5) 22.1 (3.9)* 25.3 (7.7) 27.5 (5.8)* <0.001

Gender

Male *n* (%) 68 (46) 3 (19) 30 (49) 35 (52) 0.97

Female *n* (%) 77 (54) 13 (81) 31 (51) 32 (48) 0.97

SMI (kg/m^2^) 6.3 (1.8) 5.0 (1.4)* 6.2 (2.6) 6.8 (1.8)* 0.002

S-cystatin C (mg/L) 0.96 (1.7) 1.1 (.2)* 1.02 (0.8) 0.88 (0.1)* <0.001

S-creatinine (ųmol/L) 57.6 (23.7) 58.8 (26.9) 62.0 (23.0) 54.0 (23.6) 0.153

Measured clearance (mL/min) 87.8 ± 22.1

(40-155)

MDRD (mL/min) 176.7 (167.3) 150.1 (200.6) 160.1 (160.9) 194.1 (163.4) 0.153

CKD-EPI (mL/min) 124.4 (36.2) 96.3 (27.2)* 116.8 (35.5) 137.7 (33.4)* <0.001

eGFR CysC (mL/min) 110.8 (24.9) 84.5 (15.4)* 100.7 (16.0)* 126.5 (19.3)* <0.001

eGFR CysC+CKD-EPI (mL/min) 119.0 (26.0) 90.4 (18.8)* 110.8 (23.0)* 133.2 (21.3)* <0.001

Diagnosis (%)

Myotonic dystrophy 1 93 (64) 14 (87) 48 (79) 31 (46)

Duchenne and Becker 6 (4) 0 (0) 4 (7) 2 (50)

muscular dystrophy

Fa~~s~~cioscapulohumeral 19 (13) 0 (0) 3 (5) 16 (24)

muscular dystrophy (FSHD)

Limb-girdle muscular dystrophy 19 (13) 2 (13) 6 (10) 11 (16)

Spinal muscular atrophy (SMA) 8 (6) 0 (0) 0 (0) 7 (10)

Results expressed as mean ±SD. Diagnosis expressed as n (%). Abbreviations: SMI, skeletal muscle index; FSHD, Fa~~s~~cioscapulohumeral muscular dystrophy; Limb-girdle, Limb-girdle muscular dystrophy; SMA, Spinal muscular atrophy, eGFR, estimated GFR, CKD-EPI, Chronic Kidney Disease Epidemiology Collaboration; MDRD, Modification of Diet in Renal Disease [31,1]

**Supplementary Table 2.** Correlations between estimated (eGFR) and kidney function (clearance) (n=145).

**Unadjusted clearance Adjusted clearance^a^**

MDRD (mL/min) 0.12 (-0.07, 0.30) 0.20 (0.04, 0.40)

CKD-EPI (mL/min) 0.43 (0.28, 0.57) 0.47 (0.34, 0.60)

eGFR CysC (mL/min) 0.67 (0.56, 0.77) 0.57 (0.43, 0.70)

eGFR Cys C+CKD-EPI (mL/min) 0.62 (0.50, 0.72) 0.58 (0.43, 0.70)

^a^Adjusted for age, gender, smoking status and muscle mass (SMI, skeletal muscle index). MDRD; modification of diet in renal disease study group, CKD-EPI; chronic kidney disease epidemiology collaboration, eGFR CysC; Cystatin C-based estimated GFR, eGFR CysC+CKD-EPI; combined Cystatin C-based and CKD-EPI (mean values), see methods section. 95% confidence intervals (95% CI) based on bootstrap in parentheses. All correlations were significant, p<0.05.

**Supplementary Table 3.**

Comparison of the performance (bias and accuracy) of estimated GFR equations (eGFR) by measured kidney function (clearance) overall and at different levels (n=145).

**Different levels of kidney function (clearance) (mL/min)**

**Overall (n=145) 30-59 (n=16) 60-89 (n=61) ≥90 (n=67)**

***Bias^a^(mL/min)***

MDRD 35.2 (26.7, 44.5)* 47.5 (15.5, 80.5) 27.5 (18.3, 42.5)* 39.5 (27.3, 48.3)*

CKD-EPI 30.1 (27.6, 36.4)* 42.9 (20.7, 53.8) 33.3 (28.1, 37.1)* 28.3 (19.1, 35.6)*

**eGFR CysC 23.6 (21.1, 25.5) 27.7 (23.4, 40.6) 24.8 (21.1, 28.3) 17.9 (12.4, 24.2)**

eGFR CysC 28.1 (25.0, 32.4)* 34.8 (25.2, 51.5) 30.6 (23.8, 37.2)* 24.5 (19.0, 29.8)*

+ CKD-EPI

***Accuracy (P10)^b^ (%)***

MDRD 16.6 (11.0, 23.4) 0.0 (0.0, 0.0) 19.7 (9.7, 30.0) 17.9 (8.8, 27.8)

CKD-EPI 9.0 (4.8, 14.5)* 0.0 (0.0, 0.0) 8.2 (1.7, 15.5) 11.9 (4.8, 20.7)*

**eGFR CysC 18.1 (11.8, 25.0) 0.0 (0.0, 0.0) 11.7 (3.8, 21.2) 28.4 (17.5, 40.0)**

eGFR CysC 11.8 (6.9, 17.4) 0.0 (0.0, 0.0) 1.7 (0.0, 5.4) * 23.9 (14.3, 35.1)

+ CKD-EPI

***Accuracy (P30) ^b^ (%)***

MDRD 37.2 (29.0, 45.5) 18.8 (0.0, 40.0) 42.6 (31.1, 54.8) 37.3 (25.4, 50.0)*

CKD-EPI 33.8 (26.2, 41.4)* 0.0 (0.0, 0.0) 27.9 (17.5, 41.1) 47.8 (34.9, 59.7)*

**eGFR CysC 48.3 (40.0, 55.9) 0.0 (0.0, 0.0) 39.3 (27.0, 50.9) 67.2 (55.4, 78.6)**

eGFR CysC 39.3 (31.7, 46.9)* 0.0 (0.0, 0.0) 29.5 (18.5, 41.8) 58.2 (46.7, 70.3)

+ CKD-EPI

a) Bias was assessed the mean difference (eGFR-measured clearance) with negative values indicating lower eGFR than measured clearance (underestimation of kidney function) and positive values indicating overestimation.

b) Accuracy is defined as the proportion of eGFRs within ±>30%v(P30) and within ±>10% (P10) of measured clearance (95% CI).

CKD-EPI, Chronic Kidney Disease Epidemiology Collaboration; MDRD, Modification of Diet in Renal Disease.

*p=<0.05 vs eGFR CysC

Supplementary figures 2 a-d.

a) Bland-Altman-plots of the differences between estimated GFR (eGFR Cys C) and measured iohexol clearance. Kidney function (measured clearance) levels as indicated; 38-59, 60-89 and ≥90 mL/min.

b) Bland-Altman-plots of the differences between estimated GFR (eGFR CKD-EPI) and measured iohexol clearance. Kidney function (measured clearance) levels as indicated; 38-59, 60-89 and ≥90 mL/min.

c) Bland-Altman-plots of the differences between (eGFR CysC+CKD-EPI) and measured iohexol clearance. Kidney function (measured clearance) levels as indicated; 38-59, 60-89 and ≥90 mL/min.

d) Bland-Altman-plots of differences between (eGFR MDRD) and measured iohexol clearance.

Kidney function (measured clearance) levels as indicated; 38-59, 60-89 and ≥90 mL/min.

Supplementary figure 2a


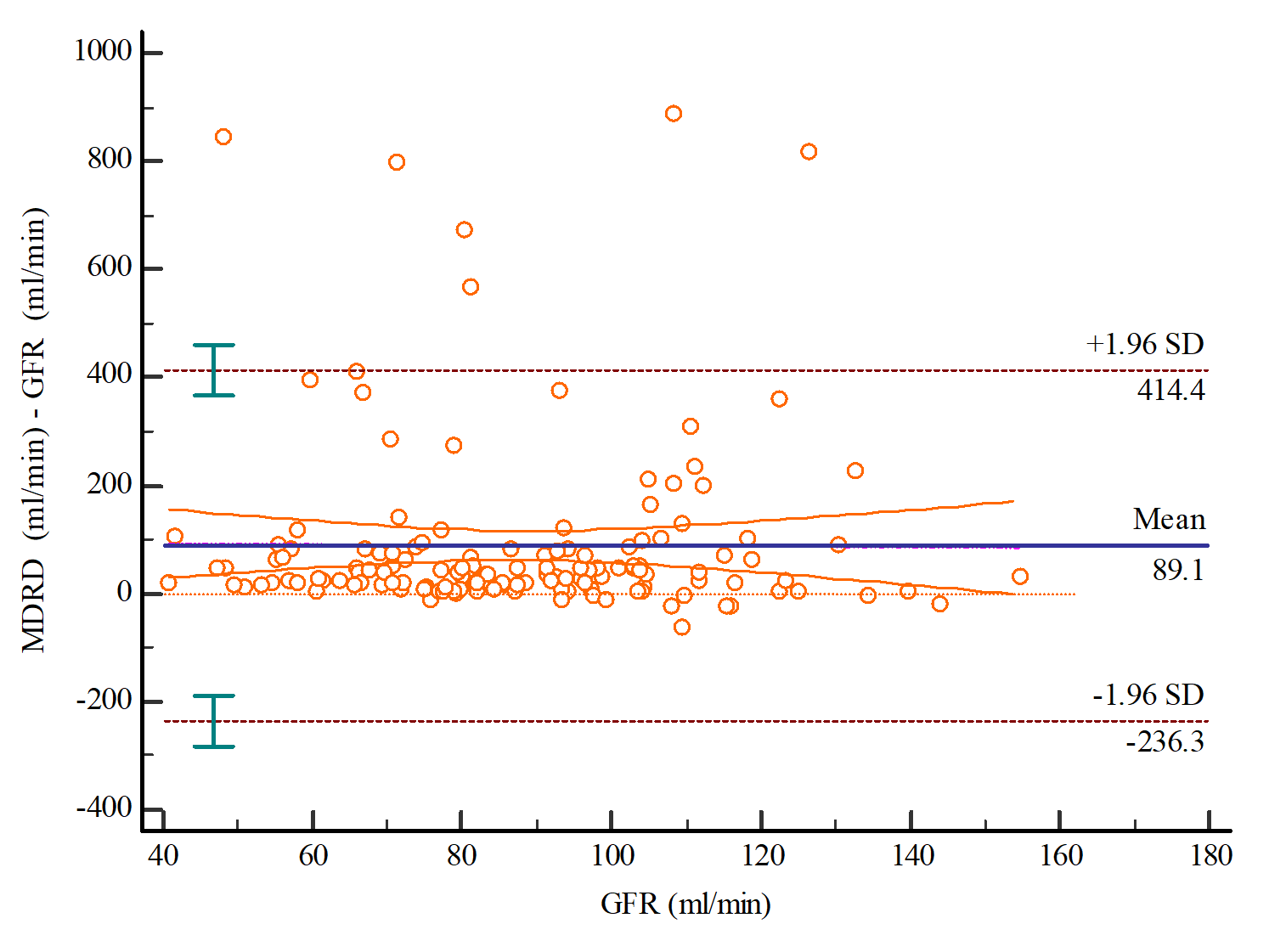


GFR=measured iohexol clearance

Supplementary figure 2b


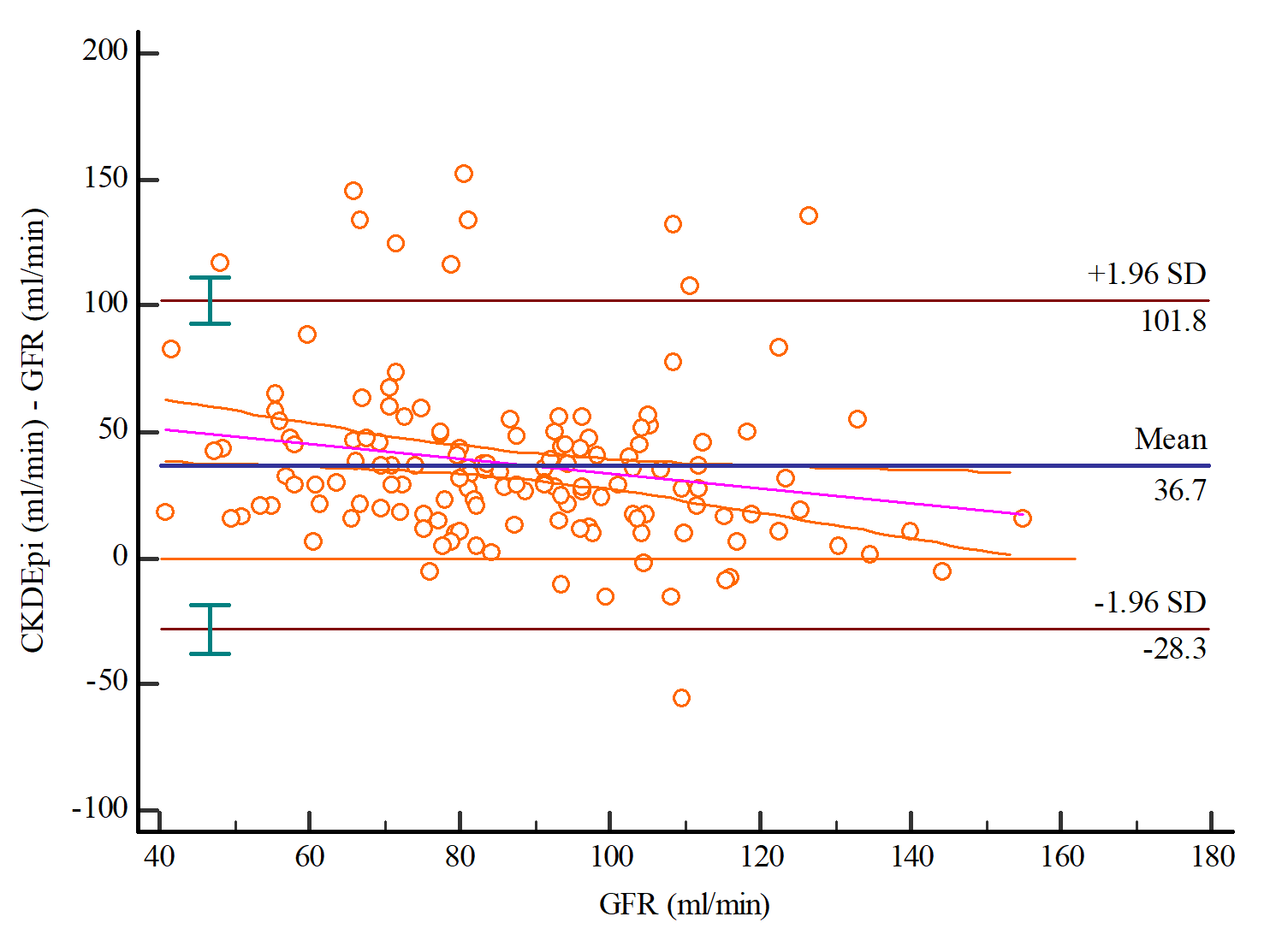


GFR=measured iohexol clearance

Supplementary figure 2c


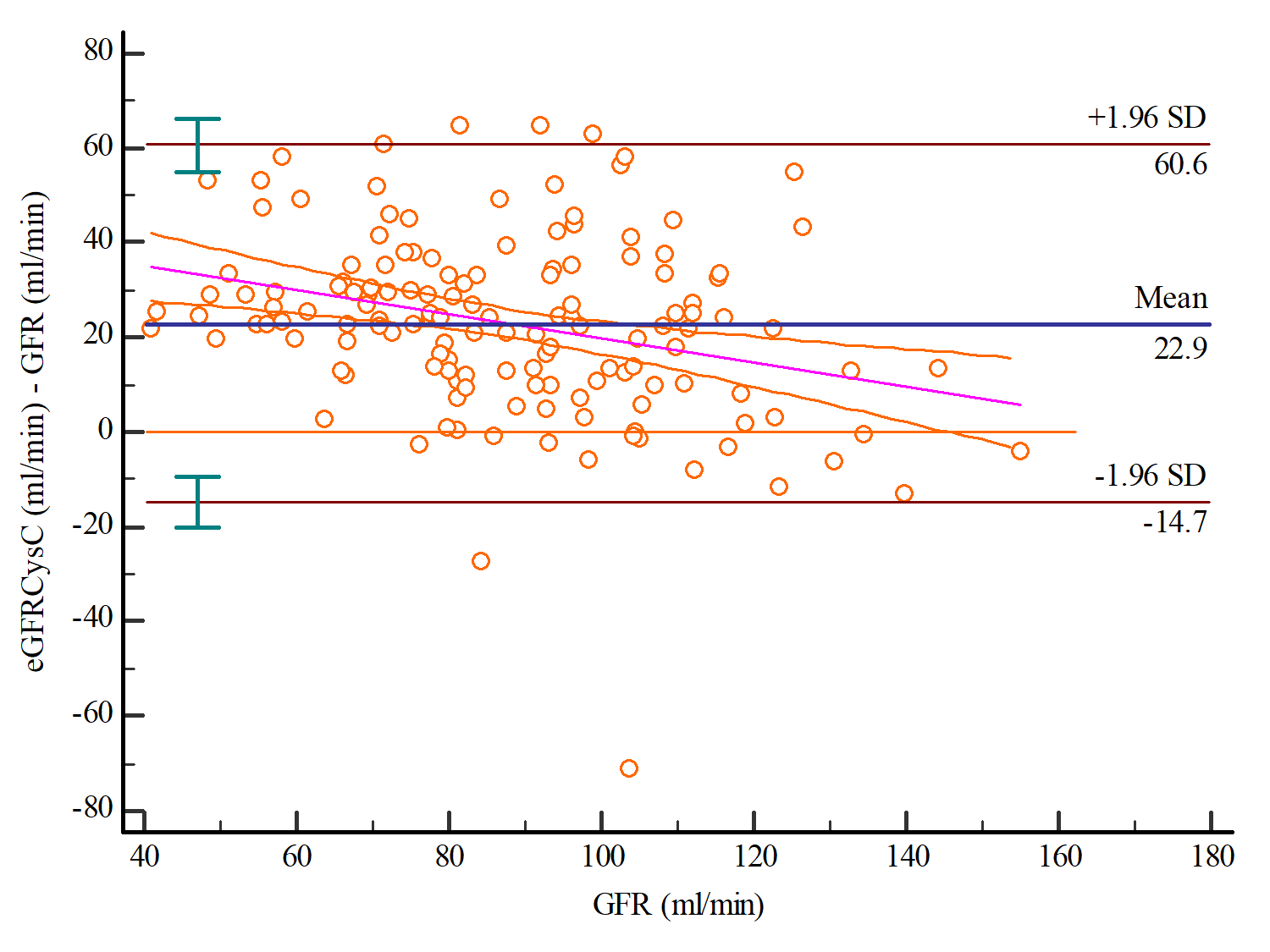


GFR=measured iohexol clearance

Supplementary figure 2d


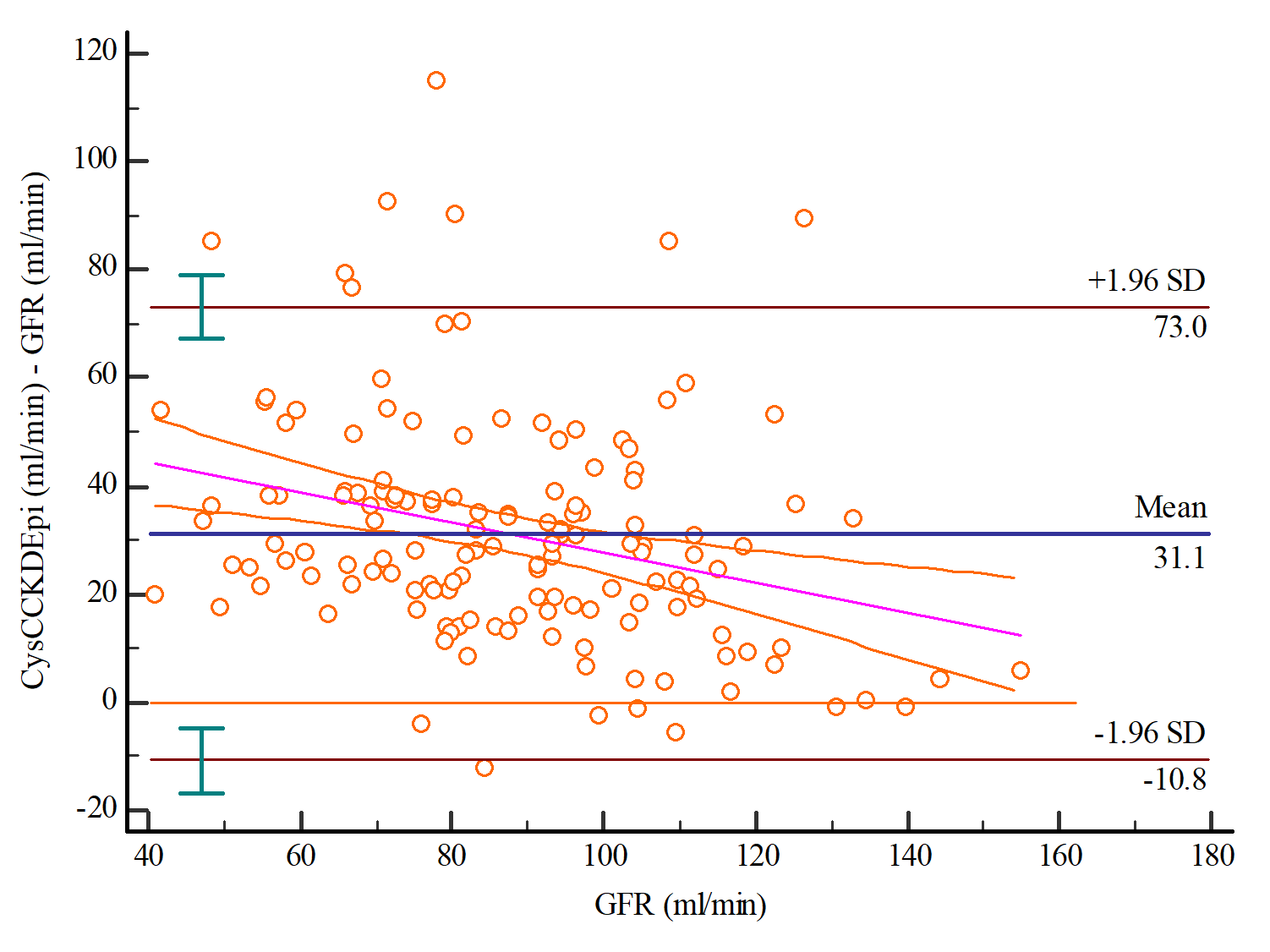


GFR=measured iohexol clearance
